# M-plasty for Full-Thickness Skin Graft Donor Site

**Published:** 2015-09-30

**Authors:** Morioka Daichi, Ohkubo Fumio, Sato Nobuhiro

**Affiliations:** Department of Plastic Surgery, Showa University, Hatanodai, Shinagawa-ku, Tokyo, Japan

**Keywords:** burn, M-plasty, full-thickness skin graft, donor site morbidity, scar contracture

**Figure:**
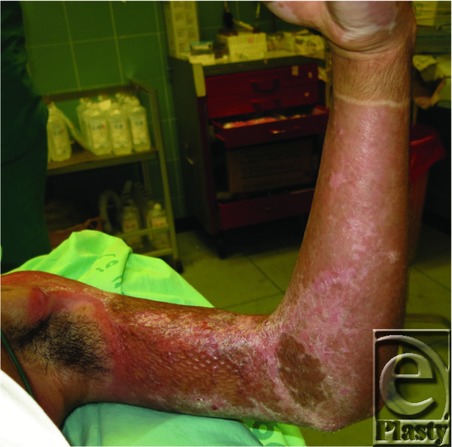


## DESCRIPTION

An 18-year-old man suffered deep dermal burns on his chest, back, and left arm after an explosion and underwent mesh-skin grafting of the affected areas over several operations. The patient came to us with a postburn joint contracture of the left elbow.

## QUESTIONS

1. **How do you harvest donor skin for a full-thickness skin graft?**
2. **What is M-plasty typically used for?**
3. **What is the technique for M-plasty for full-thickness skin graft donor site?**
4. **When compared with an ordinary fusiform skin harvest, what are the advantages and disadvantages of using M-plasty for the donor site?**

## DISCUSSION

**Figure 1:**
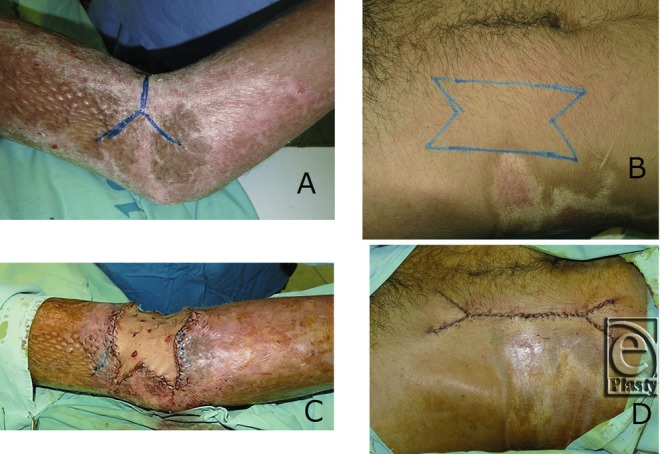
Preoperative view of postburn scar contracture on the left elbow. (a) A double-Y incision is designed. (b) A double M-plasty designed for a full-thickness skin donor site on the abdomen. The harvested skin was identical to the defect on the elbow. (c) Full-thickness skin graft performed without trimming after double-Y release of the contracture. (d) Two-layered closure with nylon sutures of the donor site. The length of scar is shortened, minimizing dog ears on the edge of the scar.

Full-thickness skin graft is primarily used for wounds that cannot be closed, defects after tumor resection, and scar contracture release. Full-thickness donor skin is usually harvested according to a template previously marked on the recipient site. Ideally, the donor site is closed as a linear scar along a relaxed skin tension line. In most cases, however, 2 triangular pieces of tissue are sacrificed when converting a circular, square, or irregular skin defect to a standard fusiform shape prior to closure. A double-Y release is often performed for postburn joint contractures.^1^ Although a fusiform full-thickness skin harvest for the double-Y defect wastes nearly 50% of the edge of harvested skin,^2^ donor site skin that is harvested with double M-plasty can be used without trimming and is directly closed as shown in Figure 1.

There have been many techniques described for avoiding skin protrusions (dog ears) after excision of tumors.^3^ One such technique, M-plasty, is frequently used for excision of skin tumors because it allows for more scar length shortening than the conventional fusiform excision, resulting in a linear scar.^2^^,^^3^ Several authors have reported modifications of M-plasty^4^ and similar techniques based on the same principle.^5^ However, almost all of these procedures are described for tumor excision.

The M-plasty can be performed at one or both sides of the wound, considering the aesthetic boundaries involved and the size and shape of the recipient defect. Lines designing the M-plasty are drawn from each side of an isosceles triangle or fusiform incision to the midpoint of the edge of the donor site. While a fusiform skin excision on convex surfaces such as the upper arm often leaves unpleasant dog ears on the edge of the scar, double M-plasty skin harvesting minimizes the dog ear. Although many reports^2^^,^^3^ state that the apical angle of the M-plasty must be up to 30°, dog ear deformities usually are not present even with a 45° angle in our experience. The donor site closure should be started from the widest point (usually in the middle of the defect). When skin tension is found, the circumference of the defect should be undermined at an appropriate depth. Undermining the tip of the “M” is not necessary and may result in ischemia of the tip.

**Figure 2:**
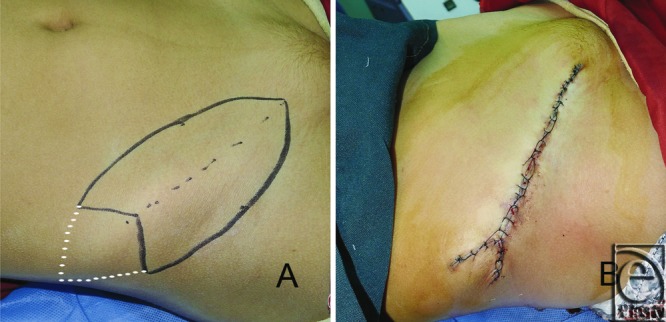
(a) A single M-plasty designed for full-thickness skin donor site on the right groin. (b) Two-layered closure with nylon sutures. The white dotted line indicates fusiform skin harvesting. The length of the scar is shortened, avoiding a scar on the iliac crest.

The groin is most frequently used for extensive defects. Skin harvesting with M-plasty along the inguinal crease can shorten scar length and avoid scar extension onto the iliac crest (Fig 2) or unfavorable damage to the hair-bearing skin. The only disadvantage of M-plasty harvest is that a 3-cornered intradermal stitch through the tip of the “M” may be somewhat difficult for less experienced surgeons. We have performed M-plasty for the harvest and closure of full-thickness skin graft donor sites for 11 patients since 2011. The donor sites included the upper arm, the groin, and the abdomen. Recipient site excisions were performed for basal cell carcinoma of the nose, hairy nevi of the cheek, and postburn contractures of the elbow joint and the neck. There were no serious postoperative complications.

In summary, when compared with a fusiform incision design, adaptation of the M-plasty principle allows for wide skin harvesting with a short line of incision and spares tissue that would otherwise have to be sacrificed to remove dog ears. Because it is relatively easy to perform and results in cosmetically acceptable scars, the M-plasty skin harvest is a welcome addition to the dermatologic surgeon's armamentarium.

## References

[1] Section III, Neligan PC (2012). Burns surgery. Plastic Surgery, Vol. 4. Lower Extremity, Trunk and Burns.

[2] Webster RC, Davidson TM, Smith RC (1976). M-plasty techniques. J Dermatol Surg.

[3] Asken S (1986). A modified M-plasty. J Dermatol Surg Oncol.

[4] Krishnan RS, Donnelly HB (2008). The nested M-plasty for scar length shortening. Dermatol Surg.

[5] Robbins TH (1976). The “crown” excision of facial skin lesions. Plast Reconstr Surg.

